# Olea Europaea Geminivirus: A Novel Bipartite Geminivirid Infecting Olive Trees

**DOI:** 10.3390/v13030481

**Published:** 2021-03-15

**Authors:** Michela Chiumenti, Claudia Greco, Angelo De Stradis, Giuliana Loconsole, Vincenzo Cavalieri, Giuseppe Altamura, Stefania Zicca, Pasquale Saldarelli, Maria Saponari

**Affiliations:** 1 Institute for Sustainable Plant Protection, CNR, Via Amendola 122/D, 70126 Bari, Italy; claudia.greco@ipsp.cnr.it (C.G.); angelo.destradis@ipsp.cnr.it (A.D.S.); vincenzo.cavalieri@ipsp.cnr.it (V.C.); giuseppe.altamura@ipsp.cnr.it (G.A.); stefania.zicca@ipsp.cnr.it (S.Z.); pasquale.saldarelli@ipsp.cnr.it (P.S.); maria.saponari@ipsp.cnr.it (M.S.); 2 Dipartimento di Scienze del suolo, della Pianta e degli Alimenti, University of Bari “Aldo Moro”, Via Amendola, 165/A, 70126 Bari, Italy

**Keywords:** high-throughput sequencing, olive trees, total DNA library, *Geminiviridae*, small RNA, ssDNA-virus

## Abstract

In 2014, high-throughput sequencing of libraries of total DNA from olive trees allowed the identification of two geminivirus-like contigs. After conventional resequencing of the two genomic DNAs, their analysis revealed they belonged to the same viral entity, for which the provisional name of Olea europaea geminivirus (OEGV) was proposed. Although DNA-A showed a genome organization similar to that of New World begomoviruses, DNA-B had a peculiar ORF arrangement, consisting of a movement protein (MP) in the virion sense and a protein with unknown function on the complementary sense. Phylogenetic analysis performed either on full-length genome or on coat protein, replication associated protein (Rep), and MP sequences did not endorse the inclusion of this virus in any of the established genera in the family *Geminiviridae*. A survey of 55 plants revealed that the virus is widespread in Apulia (Italy) with 91% of the samples testing positive, although no correlation of OEGV with a disease or specific symptoms was encountered. Southern blot assay suggested that the virus is not integrated in the olive genome. The study of OEGV-derived siRNA obtained from small RNA libraries of leaves and fruits of three different cultivars, showed that the accumulation of the two genomic components is influenced by the plant genotype while virus-derived-siRNA profile is in line with other geminivirids reported in literature. Single-nucleotide polymorphism (SNP) analysis unveiled a low intra-specific variability.

## 1. Introduction

Olive (*Olea europaea* L.) is an important crop in Italy in terms of olives and oil productions, cultivated area, and cultural heritage. In 2018, the estimated area covered by this crop reached over 1 million hectares with a yield of about 1.6 t/ha (FAOSTAT, 2020) and Italy ranks first in olive oil consumption. As for other vegetatively propagated crops, olive can be affected by several viruses and virus-like diseases. Sixteen (16) viruses belonging to eight genera have been described on olive so far [1,2,3], most of them from symptomless plants or detected in a very limited number of trees [4,5,6,7,8,9,10,11], and in most cases with no clear evidence of their etiological involvement in a disease. An exception is olive leaf yellowing-associated virus (OLYaV; [12]), whose detection in plants with yellowing and woody cylinder symptoms strongly advocate its involvement in the disease.

The advent of high throughput sequencing (HTS) technology represents an authentic revolution in the field of virology. Indeed, in contrast with classical molecular and serological techniques, HTS can produce sequence data of almost every putative viral agent present in a sample without the need of any knowledge of the infectious agents [13,14]. Thus making the characterization of the “virome” of a plant easy and leading to the discovery of numerous viruses mostly causing latent infections, and providing novel insights in viral candidate pathogen discovery and viral ecology [13].

The use of HTS also makes possible the characterization of the fine-tuning interactions in the plant-virus system. It is well known that plants can activate a variety of defense mechanisms to counteract the infection by pathogens, among which small RNA (sRNA)-directed silencing represent a crucial biological process to regulate and silence genes both at transcriptional (by DNA methylation or histone modifications of the DNA targets) and post-transcriptional level (inactivating or degrading of target transcripts) [15,16,17,18]. Such pathways are controlled by small RNAs (sRNAs): short RNA fragments 21- to 24-nt-long, which in plants are produced by Dicer-like proteins (DCL) and their associated double stranded RNA (dsRNA) binding proteins, cleaving dsRNAs or highly structured single stranded RNA. RNA-dependent RNA polymerases (RDR) contribute to the synthesis of the dsRNA substrate diced in a specific length by specific DCLs proteins. sRNAs are then loaded in Argonaute (AGO) proteins for targeting complementary nucleic acids for post-transcriptional or transcriptional gene regulation [16,17,19]. Specifically, DCL1 and AGO1 are required mostly for the biogenesis of the micro-RNAs (21 nt); DCL2, RDR6, and DCL4 are involved in natural antisense small interfering RNAs and trans-acting siRNAs pathways, respectively. RDR2, DCL3, and AGO4, or AGO6 are required for the heterochromatic siRNAs (24 nt) implicated in the transcriptional gene silencing [20]. Considering that, viruses completely rely on host resources for their reproduction and dissemination, the sRNA silencing pathways play a fundamental role in anti-viral defense. Indeed, viruses can be either triggers or targets of the RNA silencing and the occurrence of a viral infection is regularly accompanied by the appearance of virus-derived siRNAs.

Geminivirids are plant pathogenic viruses of major importance due to their ability to cause major diseases worldwide [21,22,23]. These viruses belong to the *Geminiviridae* family and are characterized by single-stranded, circular DNA genomes ranging from 2500 to 5200 nucleotides in length and bungled into non-enveloped geminate particles. To date, nine different genera have been described for this family, *Becurtovirus, Begomovirus, Capulavirus, Curtovirus, Eragrovirus, Grablovirus, Mastrevirus, Topocuvirus,* and *Turncurtovirus*, distinguished based on their genome organization, genome-wide pairwise identity, insect vector and host range, mainly herbaceous plants [24]. Other geminivirids not yet assigned to a genus, such as citrus chlorotic dwarf-associated virus (CCDaV), mulberry mosaic dwarf-associated virus (MMDaV), apple geminivirus (AGV), grapevine geminivirus A (GGVA), Prunus geminivirus A (PrGVA) and wild vitis virus-1 [25,26,27,28,29,30] have been described on woody hosts. Where known, all geminivirids can be transmitted in persistent, circulative and non-propagative way by different types of insects (whiteflies, leafhoppers, treehoppers, and aphids) [24], depending on specific amino acidic sequences in the capsid protein [31]. Among the *Geminiviridae*, the group with the largest number of species is the genus *Begomovirus*. To date, the International Committee on Taxonomy of Viruses (ICTV) recognizes 597 species, infecting dicotyledonous plants. Begomoviruses can have either monopartite or bipartite genomes, in the latter case with genomic components known as DNA-A and DNA-B. Bipartite genomes share a 200-nt-long region, which includes the origin of replication [32]. Genomic organization of monopartite begomoviruses is similar to that of DNA-A of bipartite ones. Generally, DNA-A and monopartite begomoviruses have open reading frames (ORFs) encoding, in the virion strand, the coat protein (AV1/V1) and the AV2/V2 protein putatively involved in virus movement, while on the complementary strand are found the replication-associated protein (AC1/C1), a transcriptional activator protein (AC2/C2), a replication enhancer protein (AC3/C3), and the AC4/C4 protein [33]. In bipartite begomoviruses, DNA-B encodes a nuclear shuttle protein (BV1) on the virion-sense strand and on the complementary-sense strand the movement protein (BC1) [34,35]. Based on their geographical distribution, a further grouping can be identified in this genus distinguishing Old World (OW) and New World (NW) begomoviruses. In the OW (Africa, Asia, Australasia, and Europe) group, most begomoviruses are monopartite, with a few having a bipartite genome, in both cases containing an AV2/V2 ORF. The NW (America) group gathers almost exclusively species with bipartite genomes and not encoding any AV2/V2 protein [36].

We report here the discovery by HTS of a novel geminivirid from olive trees (*Olea europaea* L.) with peculiar molecular and phylogenetic features. This novel virus, provisionally named Olea europaea geminivirus (OEGV), has a bipartite genome with the DNA-A genomic organization similar to that of NW begomoviruses. Its DNA-B shows a unique organization including two proteins: on the virion-strand a BL1 begomovirus-related protein and a completely divergent undescribed second protein on the complementary-strand. The survey conducted in different olive orchards in Apulia revealed this virus is widely present even though it could not be associated to any symptom. Furthermore, the evolutionary relationship of OEGV with other known members of the family *Geminiviridae* indicated that OEGV is a virus with unique genome features, possibly representing a new genus in this family.

## 2. Materials and Methods

### 2.1. Plant Materials

#### 2.1.1. NGS Libraries and Identification of Gemini-Like Sequences

Two whole genome DNA libraries were prepared at the end of 2013 with the primary objective of sequencing the full genome of the plant pathogenic bacterium *Xylella fastidiosa* decimating olive trees in the Apulia region (southern Italy). To this end, two olive trees were sampled: one Xylella-infected tree was selected in the outbreak area and used to recover the sample identified as PC, and one *Xylella*-free tree, identified as “OS”, was sampled in the repository of olive cultivars maintained at the research center “Centro di Ricerca, Sperimentazione e Formazione in Agricoltura”, Palagiano, Italy. The *Xylella*-infected tree belonged to the cultivar “Ogliarola”; the non-infected tree “OS” belonged to the cultivar “Leccino”. Total DNA was recovered from mature twigs using cetyl trimethylammonium bromide (CTAB) based extraction protocol, followed by an RNAse treatment. For the DNA extracts, library construction and sequencing with Illumina technology in a 100 bp paired end sequencing format were outsourced. Further viral genomic analyses were performed on the tree “OS”, to confirm in vivo the occurrence of the contigs assembled from the libraries and to complete its genomic sequence by conventional PCR. To this end, total DNA was recovered using a CTAB-based extraction protocol from 200 mg of olive leaf petioles [37]. This template was used for PCR and inverse-PCR experiments with 8 and 12 different primers, targeting DNA-A and DNA-B, respectively (Table S1). TaKaRa SpeedSTAR DNA polymerase (Takara Bio Europe, Saint-Germain-en-Laye, France) was used for PCR with a two-step amplification protocol. PCR amplicons of the expected sizes were purified and directly sequenced (Macrogen Europe, Amsterdam, the Netherlands).

#### 2.1.2. Occurrence of the Novel Virus in Olive Groves

We selected a total of 55 DNA extracts representing different geographic areas and provinces of the region: Bari (15), Brindisi (20), and Taranto (20). These DNA extracts were recovered from plant tissues consisting of leaf petioles and small portions of twigs, homogenized using the semi-automated homogenizer Homex 6 (Bioreba, Reinach, Switzerland) with 5 mL of Food Lysis Buffer (Qiagen, Venlo, The Netherlands) and then processed following the manufacturer’s instructions of the DNeasy mericon Food Kit (Qiagen Venlo, The Netherlands). The recovered total DNA was then subjected to conventional PCR using the diagnostic primers designed on DNA-A and DNA-B (Table S1) and following standard amplification conditions.

#### 2.1.3. Assessment of the Small RNA Profiles

Six libraries of small RNA were already available from a collaborative project between our Institute and the Dipartimento di biologia e chimica agro-forestale ed ambientale of the University of Bari (Italy). These libraries were prepared in late 2012 from olive trees using TruSeq Small RNA Sample Preparation kit (Illumina, Eindhoven, The Netherlands). The accessions, selected from the same repository mentioned above, belonged to the cultivars Toscanina (TS), Oliastro (OL), and Leucocarpa (LC). Total RNAs were extracted using a guanidine-based extraction protocol and the sRNAs of 19–26 nt in length were enriched as described by Giampetruzzi et al. [38]. Small RNA fractions were recovered both from fruits and from leaves collected in late autumn from each of the selected trees. Small libraries were sequenced in a 50 bp single read format on HiScan SQ (Illumina, Eindhoven, The Netherlands).

### 2.2. Bioinformatics Analysis

Libraries were quality controlled and adapter sequence were removed using FastX toolkit. DNA reads with QC > 20 were used for assembly with EDENA V3.131028 [39] and annotated by BLASTn and BLASTx searches, adopting respectively cut-off e-values thresholds of 10^−6^ and 10^−4^.

In silico validation of conventional sequencing results and sRNA characterization was carried through mapping alignments with bowtie2 [40] and PatMaN [41] using reassembled genomic fragments as reference sequences. Secondary structures of the common regions were predicted using the mFold webserver [42]. sRNAs were plotted using a custom-made R script, while 5′ nucleotide analysis was performed with MISIS program [43]. Variant calling was obtained using bowtie2 alignments submitted to vcftools pipeline [44].

### 2.3. Genome Organization and Homology Searches for OEGV Genes

PCR products obtained through conventional Sanger sequencing were assembled using the BioEdit 7.0.9 program CAP-contig assembly tool [45]. ORFs were identified with ORFfinder (https://www.ncbi.nlm.nih.gov/orffinder/ (accessed on 29 September 2020)) and conserved domains were characterized using SMART tool [46], BLASTx [47] and CCD conserved domain database with CD-Search [48].

Identity matrices were obtained using the MUSCLE option in SDT v1.2 [49]. Alignments for nucleotide and amino acid homology were done with the MUSCLE algorithm [50] embedded in MEGAX [51]. Phylogenetic relationships among members of geminivirids were evaluated using full-length nucleotide sequences with the neighbor-joining method in MEGAX program [51] with 1000 replicates of bootstrap as already described by the ICTV Taxonomy study group [24]. Maximum-likelihood phylogenetic trees were inferred from Rep, CP, and BL1 genes of representative nucleotide isolate sequences of viruses from genera in the family *Geminiviridae*, applying the GTR + G, GTR + G + I and TN93 + G substitution models, respectively.

### 2.4. Rolling Circle Amplifications and Southern Blot Assay

Total DNA was extracted using the CTAB protocol [37]. One µL of CTAB total DNA extract was used for rolling circle amplification (RCA) with Illustra TempliPhi Amplification kit (GE Healthcare Life Science, Chicago, IL, USA) according to the manufacturer’s protocol. RCA products were digested in 50 µL with the specific one-cut restriction enzymes, cutting outside the complementary probe sequence, BamHI (G/GATCC, position on the genome: 632–637), NcoI (C/CATGG, position on the genome: 733–738) and PstI (C/TGCAG, position on the genome: 1145–1150) for 3 h at 37 °C.

DIG-labelled DNA probe was synthesized with the PCR DIG Probe Synthesis kit (Roche, Monza, Italy), using as template the DNA-A amplicon obtained with the diagnostic primers (AC1-for/AC1-rev; Table S1). For Southern blot analyses, the gel was blotted on Amersham Hybond-N+ membrane (GE Healthcare Life Science, Chicago, IL, USA) that was pre-hybridized at 44 °C for 30 min in DIG Easy Hyb solution (Roche, Monza, Italy). Hybridization was run overnight at 44 °C with the DIG-labelled OEGV-DNA-A-specific probe. The membrane was subjected to chemiluminescent detection using ChemiDoc apparatus (Bio-Rad, Hercules, CA, USA).

## 3. Results

### 3.1. High-Throughput Sequencing Results

The high-throughput sequencing of the two DNA libraries, OS and PC, yielded 14,548,305 and 14,166,962 raw paired reads respectively. A total of 3628 and 4263 contigs having the size of 151–73,533 and 151–68,485 nucleotides were obtained from the OS and PC libraries, respectively. BLASTx search of these contigs indicated the presence of two virus-derived DNAs sharing similarity with members of the *Geminiviridae* family in each of the two libraries. Among these annotated sequences, two contigs of 2824- and 2566-nt-long, identified respectively in OS and PC, were homologous to DNA-A of bipartite begomovirus. Similarly, additional two contigs of 2579- and 2854-nt-long, retrieved respectively in OS and PC datasets, shared similarities to begomoviral DNA-B.

**Figure 1:**
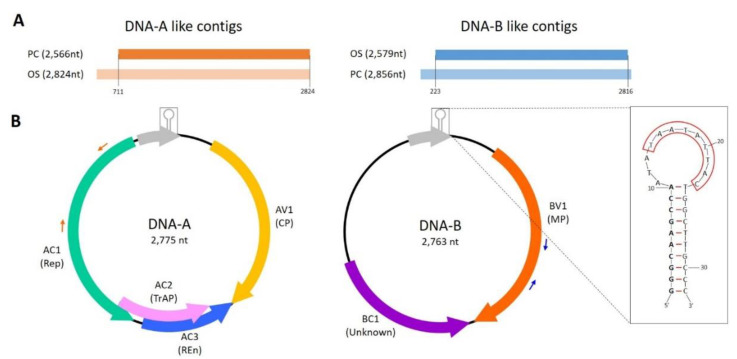
Graphical representation of the pairwise alignment between homologous contigs retrieved in the two DNA libraries. Darker shade-colored bars represent the smaller contigs, lighter colored bars stand for the longer contigs. The library of origin of each contig has been indicated with the library names, PC and OS. Numbers within parentheses indicates the nucleotide contig length. Vertical black lines delimit the aligned regions, while the numbers at the bottom of the vertical lines indicate the coordinates of the aligned regions on the longer contigs. (**B**) Genomic organization of Olea europaea geminivirus (OEGV). Information of the size of each genomic component is given. Open reading frames (ORFs) are indicated by arrows and labelled (V) or (C) if encoded on the virion or complementary-sense strand, preceded by component designation (**A** or **B**). Corresponding protein products are specified within parenthesis. Grey bold arrows indicate the putative ORF identified in the intergenic region. A small hairpin structure in grey, indicates the position of the stem-loop. A detail of such hairpin is represented in box on the right of the DNA-B genomic component. The nonanucleotide, origin of replication is framed in red. Small red and blue arrows represent the directions and position of the diagnostic primers used in this study.

Pairwise alignment of putative DNA-A-like contigs revealed that DNA-A PC aligned from position 771 until the end of the DNA-A OS contig. In this region, only two polymorphisms were observed out of the 2054 nts aligned. In the case of DNA-B-like contigs, pairwise comparison revealed that the 2579 nts of the DNA-B OS were aligned to the DNA-B PC contig from position 273 to 2816, with 100% of sequence identity (Figure 1A). A more accurate analysis of the sequence on the two 2.8 kb contigs (DNA-A OS and DNA-B PC) disclosed that both were greater-than-full-length circular fragments and that the actual length of the two genomic DNAs was 2775 nts for DNA-A-like and 2763 nts for DNA-B-like.

To further support the data retrieved in silico and completely re-sequence the viral genomes, diverse primer sets scattered along the two genomic components were designed and used in direct and reverse PCR experiments (Table S1). Obtained amplicons were submitted to direct Sanger sequencing from both ends. These sequences were reassembled using CAP-contig, confirming the nucleotide sequences constructed in silico.

Since begomoviruses could be associated with satellite DNAs, whose unique sequence homologies with their helper virus are the stem-loop and the nonanucleotide origin of replication (5′-TAATATTAC-3′; [52]) a specific BLASTn search of this nucleotide fragment has been done on the *de novo* assembled contigs of both OS and PC libraries. Such investigation resulted in no contigs other than those previously identified, thus suggesting that the viral sequences initially identified are not accompanied by any satellite DNA in the samples analyzed.

### 3.2. Genomic Features

The complete genomic sequences, DNA-A and DNA-B were investigated to identify and characterize the genomic features and the putative encoded proteins.

Search for ORFs with ORFfinder indicated that the overall sequence architecture of the DNA-A and DNA-B resembled that of NW bipartite begomoviruses (Figure 1B). Four ORFs, one in the virion-sense and three in the complementary-sense were identified in DNA-A. ORF AV1 in the virion-sense starting at nt 182 up to 949 frame +2, was predicted to encode a 255 aa coat protein (CP), sharing 30.74% sequence identity at aa level with the mastrevirus Tobacco yellow dwarf virus A (GenBank JN989443). Conserved domain analysis by NCBI-CDD tool indicated the presence of a Gemini_coat domain (pfam00844, e-value: 1.14 × 10^−14^). Three more ORFs (AC1–AC3) were identified on the complementary strand. Protein AC1 (Replicase or Rep) starts in positions 2537 to 1443, in the frame −2, encoding a protein of 364 aa sharing the highest sequence similarity with the begomovirus East African cassava mosaic virus (GenBank AJ717548) to which has a 58.26% aa identity. Domain analysis of this protein product showed the presence of two conserved signatures, the Gemini_AL1 (pfam00799, geminivirus Rep catalytic domain) and Gemini_AL1_M (pfam08283, geminivirus rep protein central domain) with e-values of 3.72 × 10^−38^ and 3.53 × 10^−23^, respectively. Protein AC2 (transcriptional activator protein or TrAP) starts at 1,531 and end at position 1,073, in the frame −3, which encodes a 152 aa product having a 36.24% aa identity to AC2 of the begomovirus Merremia mosaic virus (GenBank NC_007965.1) and containing the conserved domain Gemini_AL2 (pfam01440, e-value 1.84 × 10^−12^), which is involved in the transactivation expression of coat protein (CP) gene and movement protein (MP). Finally, AC3 (replication enhancer protein or REn) starts at 1386 and ends at position 946, frame −1, and encodes a protein of 146 aa showing the 33.33% of amino acidic sequence identity with the begomovirus Tomato leaf curl Sudan virus (GenBank EF110891). This protein is characterized by the conserved domain Gemini_AL3 (pfam01407, involved in viral replication) with e-value 6.36 × 10^−11^. Akin to the NW begomoviruses, it was not possible to identify any AV2 protein on the virion-sense of DNA-A.

Only two ORFs were identified in the B genomic component. Contrary to other NW begomoviruses, the putative movement protein (MP or BV1) is encoded on virion sense, starting at position 309 and ending at position 1199 in the frame +3. The corresponding protein 296-aa-long product shares the 39.50% of sequence identity with the begomovirus Jatropha curcas mosaic virus (GenBank GQ924761) and contains the distinctive Gemini_BL1 domain (pfam00845, e-value 6.41 × 10^−57^; protein involved in systemic infection of the host and for viral replication and encapsidation). The other ORF, BC1, in frame −2 of the complementary sense, starts at position 1,757 and stops at 1224 (177 aa). When searched either in BLASTx, in NCBI-CDD or in SMART it did not show any similarity with other proteins nor the presence of an already characterized conserved domain.

A region, common to the two DNA components has been found astride the TATA box and the nonanucleotide sequence “TAATATT/AC”, which identify the origin of DNA replication. In DNA-A, this region was defined by nucleotides 2513 to 81, whereas on DNA-B it is situated between nucleotides 2501 to 81. Pairwise alignment of these sequence fragments extracted from the two components revealed an almost total sequence identity (99.99%) with only two polymorphisms out of the total 348 nucleotides, suggesting that the two DNAs are components of the same bipartite virus. DNA folding of such conserved region (CR) using mFold web server supported the stem-loop structure of this fragment, accessed by the Rep protein as already described for other ssDNA viruses [53,54] (Figure 1B).

### 3.3. Phylogenetic Relationship with other Member of the Family Geminiviridae

Relationships between OEGV and other geminivirids were initially examined by comparison of the whole genome sequences by nucleotide pairwise alignments and identity matrices. Successively, phylogenetic analysis has been run at nucleotide level sampling representative geminiviral Rep, BL1, and CP sequences.

Following the guidelines suggested by Brown et al. [32], a BLASTn search of the DNA-A in the “non-redundant nucleotide” database has been performed to identify the 100 most similar sequences. AV1 shares the highest sequence identity with mastreviruses, the overall sequence similarity of DNA-A is mostly with begomoviruses, as all the top 100 hits belong to genus *Begomovirus* (Table S2). The full-length genome sequences of these top hits were downloaded, aligned with the MUSCLE algorithm, and pairwise compared using SDT v1.2. The obtained identity matrix (Table S2, Figure S1) revealed that OEGV sequence similarity with the other viruses ranges from 62.7 to 57.1%, well below the begomovirus species demarcation threshold of 91% [32].

**Figure 2:**
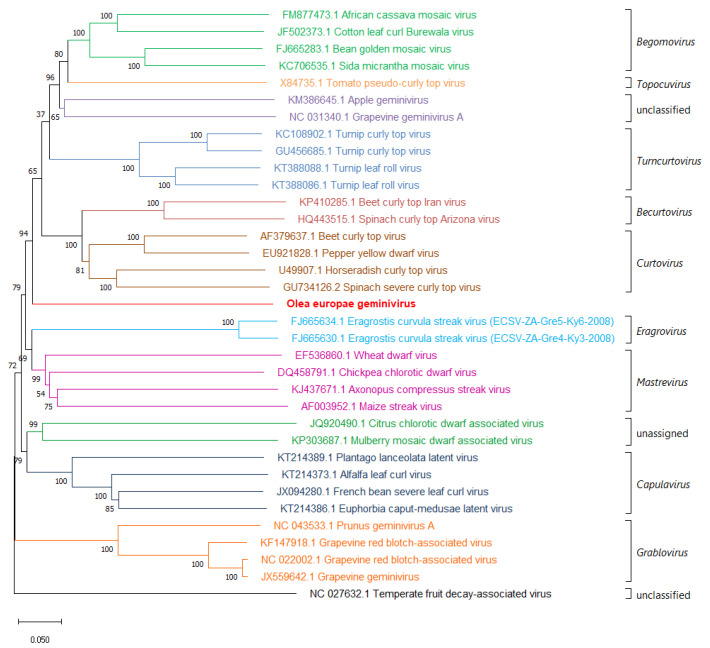
Phylogenetic trees reconstructed by the neighbor-joining method from the alignment of the full-genome nucleic acid sequence of OEGV DNA-A and representative geminivirids using MEGAX. Bootstrap values (%) for 1000 replicates are indicated on the nodes. For each virus, GenBank accession numbers and virus names are indicated in the tree. Taxonomical classification at genus level is reported on the right side of the tree, delimited by squared brackets. Horizontal bar, 0.05 substitutions per nucleotide position.

Neighbor-joining phylogenetic analysis of complete genome sequences (DNA-A sequences in the case of bipartite begomoviruses) from isolates of representative species [24] shows that OEGV clusters with the begomoviruses, topocuviruses, unclassified apple geminivirus and grapevine geminivirus A, turncurtoviruses, becurtoviruses, curtoviruses, eragroviruses, and mastreviruses, and forms a distinct branch between the curtovirus, and becurtovirus clade and the eragrovirus, and mastrevirus clade (Figure 2).

**Figure 3:**
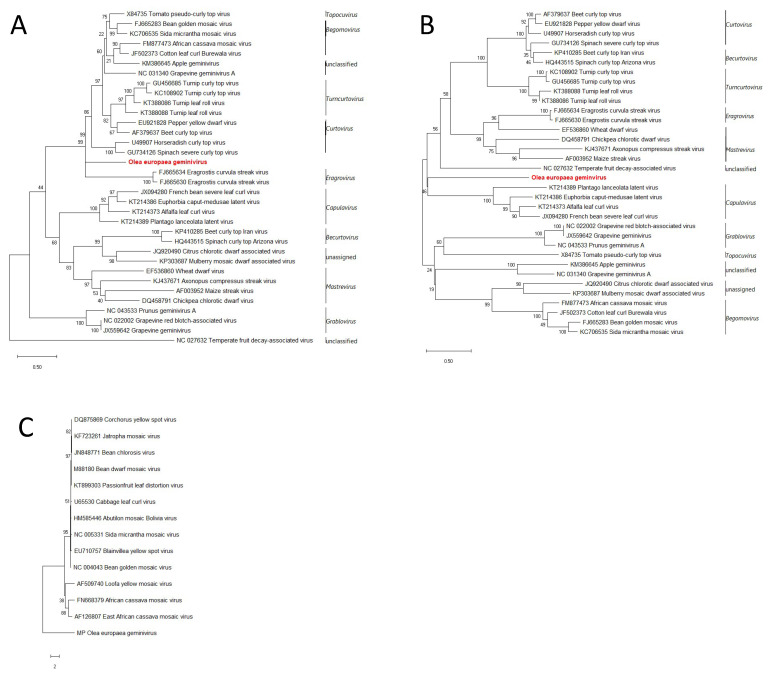
Phylogenetic trees generated by the maximum-likelihood method from the alignment of Rep (**A**), CP (**B**), and BL1 (**C**) nucleotide sequences of OEGV and members of the genera *Becurtovirus, Begomovirus, Capulavirus, Curtovirus, Eragrovirus, Grablovirus, Mastrevirus, Topocuvirus, Turncurtovirus,* and other unclassified geminivirids. Bootstrap percentages for 1000 replicates are indicated for each node. Accession numbers are given together with the virus name. Horizontal bars indicate the number of substitutions per nucleotide position. Taxonomical classification at genus level is reported on the right side of the tree, delimited by vertical lines. Olea europaea geminivirus is indicated in red.

Maximum likelihood (ML) analysis using the nucleotide sequences of Rep and CP proteins, revealed that, in both cases, OEGV clusters in separate branch compared to other genera. In the case of the Rep, OEGV clusters with the topocuviruses, begomoviruses, the unclassified apple geminivirus and grapevine geminivirus A, turncurtoviruses, curtoviruses, and eragroviruses, and forms a distinct branch between the curtoviruses and eragroviruses (Figure 3A), while its CP sequence forms a branch not correlated with any of the other clades (Figure 3B). In the case of BC1, ML phylogenetic tree revealed that OEGV-BL1-like-gene clusters as an outgroup when compared to other BL1 sequences of bipartite begomovirus (Figure 3C).

### 3.4. A Survey for OEGV in Apulian Orchards Reveals This Virus Is Widespread

To understand the distribution of the virus in olive orchards a survey has been performed. Leaf samples were collected in different areas and provinces of Apulia region (Southern Italy). Each plant sample was tested with diagnostic primers designed either on DNA-A or DNA-B (Table S1). Samples testing positive or negative with both primer sets were considered positive or negative, respectively. In Bari province, 15 samples were tested, and only two were found negative to the virus. In Brindisi province, one sample tested negative out of twenty. Finally, in Taranto province, we could find only two plants negative to the virus out of the twenty analyzed (Table S3). None of the analyzed plants showed any kind of symptom.

### 3.5. Attempts of Further Characterization by Southern Blot and Electron Microscopy

To investigate whether the viral DNA was integrated into the olive genome or replicated as an episomal form a Southern blot hybridization assay was done.

Multiple hybridization attempts were done with total DNA extracts obtained using CTAB protocol or with partial purification of viral particles. Partial purified preparations were also directly observed in electron microscopy (EM), but it was not possible to detect any gemini-like particles.

Nevertheless, these preparations tested positive in PCR using both diagnostic primer sets when checked at different final DNA concentrations and exposures in the hybridization assays. None of them produced a noticeable specific band of the expected full-length genome size, in some cases non-specific smears were visible. Thus, we decided to change approach, testing PCR positive samples in different preparations, as total DNA CTAB extracts, rolling circle amplifications (RCAs) undigested or digested with the specific DNA-A one-cut enzymes BamHI, PstI, and NcoI. Whilst a total amount of 2 ng of the DNA-A PCR amplicon used to synthesize the probe was loaded as positive control.

A total of 10 µg of 1a and 2d CTAB extracts (Table S3) together with 4 samples randomly picked among the ones tested positive in the survey were loaded on the 1% gel agarose for Southern blot hybridization. Extracts 1a and 2d were also used for the RCAs, which were loaded either undigested or digested by the three restriction enzymes mentioned above. Interestingly, although abundant DNA was visualized from the CTAB extracts on the agarose gel (data not shown), in the hybridization assay none of them showed any specific band for the virus neither as episomal DNA nor as plant genome integrated viral sequences. On the contrary, RCA products which did not show any kind of specific band on the agarose gel, exhibited a clear band of the expected DNA-A full length genome in hybridization assay. As expected, the amplicon used as positive control correctly hybridized with the probe (Figure S2).

### 3.6. Analysis of the Virus-Derived sRNAs in OEGV-Infected Olive Trees

Given the availability of sRNA libraries from previous experiments (see Section 2.1.3), we characterized the virus-derived sRNA (vd-sRNA) sequences of six sRNA libraries obtained respectively from fruit and leaf tissues of three Italian cultivars infected by OEGV (LC, TS, and OL).

The libraries were quality controlled and in Table 1 are reported the total number of reads per each library, and OEGV mapping and coverage parameters. As previously described for other geminivirid-infected small RNA datasets [55,56], the overall library profiles showed a bimodal distribution, with the most abundant peaks at 21- and 24-nt (Figure S3).

A PatMaN alignment, with default parameters, of all the reads against the two assembled reference sequences was computed. Considering the circular conformation of the genomic components of the gemini-like viruses, and to detect all sRNA mapping over or between the conventional 5′ and 3′ ends of the genome, the first 24 nts of the 5′ termini were added at the 3′ end of the linear OEGV full-length genomic components.

**Figure 4:**
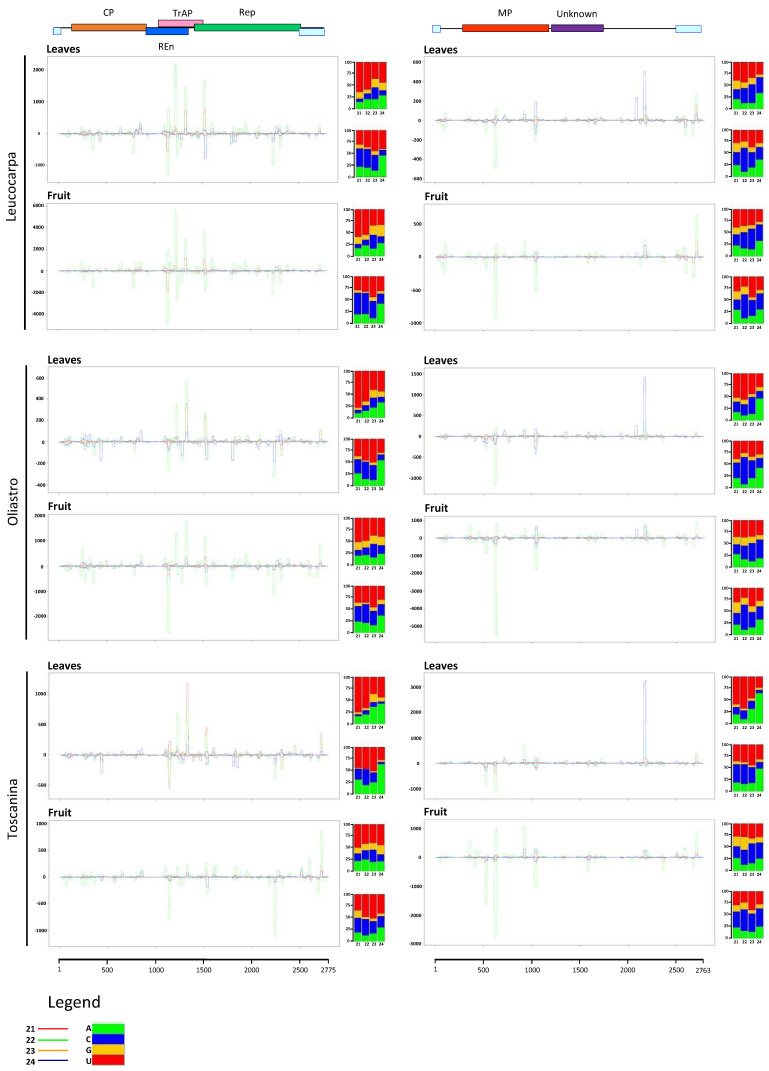
Graphical representation of the OEGV-derived sRNAs mapping on the two genomic components, DNA-A on the left and DNA-B on the right. Colored lines represent accumulation at single position of sRNAs of each size class according to the line legend showed at the bottom. Genome organization of OEGV DNA-A and DNA-B are indicated in linear form in the upper part of the panel. The ORFs encoded on the virion and complementary strand are shown together with the indication of their protein products. 5′ nucleotides of each size class have been detailed in the histogram on the right side of each size class distribution plot. Histogram bars represent the percentage of each of the four nucleotides, color code is detailed in the legend.

The two genomic components were almost fully covered by siRNAs of each sample libraries (Figure 4, Table 1). DNA-A reference sequence coverage ranged from 99.5% for LC leaves to 98.5% in sample of fruit of TS, while in the case of DNA-B the coverage percentage varied from 98.6% in fruit of OL to 97.3% in leaves of LC and fruit of TS. In all libraries, the average coverage extended from 59.8X for DNA-B in leaves of LC to 710.9X in case of fruit of LC. SRNA accumulation for the two components showed a cultivar-related relationship, as suggested by the normalized read count in reads per kilo base per million (RPKM; Table 1). Indeed, a prevalence of DNA-A accumulation was observed in LC, the two genomic components were almost equivalent in OL, while TS revealed a prevalence of DNA-B. Finally, RPKM values disclosed a general higher accumulation of OEGV-derived-sRNAs in fruit tissues rather than leaves.

Size class distribution of OEGV DNA-A mapping sRNAs revealed a prevalent peak of 22-nt species followed by 21-nt and 24-nt (Figure S3), while DNA-B mapping sRNAs showed a prevalence of 22-, followed by 24- and 21-nt.

Mapping OEGV-derived sRNAs of both polarities revealed extensive targeting of the viral genomic components. Vd-sRNA revealed a preference for certain hotspots on each of the two genomic components (Figure 4). Overlapping peaks of 21-, 22- and 24-nt sRNAs along the genomes suggest the activity of different DCLs in the biogenesis of each size class. Indeed, target nucleotide composition and/or structure may influence the activity of the diverse DCLs, involved in the different size class biogenesis, on the same genomic areas of the OEGV-derived dsRNAs. sRNAs were also unevenly distributed along the genomic components showing a slightly higher accumulation in correspondence with the AC2 protein on DNA-A and on BL1-like protein on DNA-B.

Accordingly, analysis of 5′-nucleotides of the OEGV-derived sRNAs from all the libraries disclosed a clear prevalence for 21-nt with U at the 5′ terminus in the case of positive stranded DNA-A-sRNA-derived, while a majority of 5′ A 24-nt was observed in the case of the negative polarity. Whereas for the DNA-B, a prevalence was revealed for 5′U in 22-nt and 5′C in 22-nt originated from the positive and negative polarity, respectively (Figure 4).

Since viruses generally accumulate in a single host as a cloud of closely related variants (viral quasi-species) [57,58], the intra- and interspecific variability was evaluated using a SNP analysis through vcftools on the OEGV-mapping sRNAs. The analysis revealed 17 and 8 polymorphic sites on DNA-A and DNA-B, respectively. (Table 2). Notwithstanding the presence of these polymorphisms, the ORF search on the consensus sequences showed a strong conservation of the frames and of the derived sequence proteins. During the analysis, no SNPs were detected in the common region containing the origin of replication, thus confirming the biological importance of this region.

## 4. Discussion

Over the past few years, the advent of new molecular techniques (i.e., high-throughput sequencing technologies and rolling-circle amplification) significantly broaden the current knowledge of geminivirids. Indeed, many divergent geminivirids infecting citrus, grapevine, *Jatropha multifidi*, various *Prunus* host, apple, pear, and chinaberry tree (*Melia azedarach*) [25,27,28,29,59,60,61,62,63] have been identified, expanding the host range to woody hosts. In the present study, we report the identification and characterization of a novel geminivirid infecting olive. The circular DNAs have been identified by bioinformatics analysis of two total DNA libraries obtained from two different olive tree samples and then, in vivo fully re-sequenced.

Molecular characterization of in vivo full-length sequences confirmed the genomic organization predicted in silico. Similarly to other NW begomoviruses, the genome consists of two genomic components and lacks the AV2 protein on the genomic component A. In silico analysis of DNA libraries excluded the presence of associated satellites in the analyzed olive libraries. The genomic organization of the putative DNA-A resemble that of other bipartite begomoviruses as it has AC1, AC2, and AC3 proteins on the complementary strand and the coat protein (AV1) on the virion-sense. Despite such DNA-A genomic organization, blast search of the coat protein sequence revealed its highest sequence identity with mastreviruses which are transmitted by leafhoppers rather than the whitefly-transmitted begomoviruses [64]. DNA-B is characterized by the presence of two ORFs, a novel undescribed protein with no known conserved domains on the complementary sense and a MP protein on the virion-sense. The latter, however, is commonly present on the complementary-sense strand in begomoviruses [33]. Furthermore, phylogenetic analysis of gemini-like landmark proteins—AC1, AV1, and BL1—confirmed the divergent position of OEGV which, indeed, does not group with begomoviruses nor with other approved geminiviral genera.

These findings together with a neighbor-joining tree of representative full-length genomes of all the genera in the *Geminiviridae* family and the overall nucleotide identity levels, suggest this virus could belong to a novel unclassified genus in the family, even though information on vectors and viral particles are currently missing. Indeed, the phylogenetic analysis of the full-length genome showed that this virus forms a separated branch in the superclade including becurtoviruses, curtoviruses, turncurtoviruses, topocuviruses, begomoviruses, and the novel unclassified AGV and GGVA. Phylogeny of Rep proteins shows that OEGV forms a separate clade within a cluster containing curtoviruses, turncurtoviruses, topocuviruses, begomoviruses, and AGV. In the case of the coat protein, although OEGV has the highest similarity with mastreviruses at amino acid level, maximum-likelihood analysis revealed that it clusters separately.

Albeit the field survey revealed that the virus is widely distributed in Apulian olive orchards, none of the analyzed trees showed symptoms. OEGV was not observed in Southern blot assays, probably because of the high presence of phenolic compounds in olive tissues [65] which can inhibit the hybridization reaction or due to a very low concentration of the virus in vivo. However, the full-length genome was detected using digested rolling circle-enriched templates.

Although other ssDNA virus-like sequences, putatively derived from some ancient geminivirid in the genus *Begomovirus* [66,67], have been shown to be integrated in plant genomes, Southern blot assay suggested that the high incidence of infected olives confirmed the episomal presence of the virus rather than its cointegration in the plant genome.

Attempts to purify the viral particles were ineffective, likely because of the low titer of the virus together with the high presence of polyphenolic compounds, which likely interfere with the stability of geminivirid particles.

In general, viruses exist in a given host as a combination of variants slightly differing from each other also known as quasispecies [58]. The coexistence of such variants, generated by the error-prone viral replication systems, increase virus ability to adapt to changing environment conditions and hosts [57]. The study of the intraspecific variability performed on the sRNA libraries, disclosed a low number of SNPs among the six samples analyzed, which was even lower on DNA-B component of the virus with only eight polymorphic sites compared to the 17 identified on DNA-A. Such SNPs were evenly distributed along the two genomic components of OEGV, with a possible cold spot in the putative ORF identified in the canonical intergenic region. The importance of such sequence for the viability of the virus might arise from a negative selection pressure due to the crucial role in the viral replication of the sequences and structures contained in this region [68].

Post-transcriptional gene silencing (PTGS) operates as an age-old antiviral defense mechanism in a wide range of eukaryotic organisms, including plants. Indeed, as already reported for a variety of viruses, including the ssDNA viruses cabbage leaf curl virus (CaLCuV; genus *Begomovirus*) and tomato yellow leaf curl China virus (TYLCCNV; genus *Begomovirus*) [55,56,69], geminivirids can be both trigger or target of this antiviral defense mechanism through the sRNA-guided RNA silencing. Size class distribution of sRNAs along the OEGV genomic components among the three cultivars indicated a vast majority of 22mers followed by 21mers and 24mers. This highlights the involvement of DLC2, DCL3, and DCL4, all engaged in the plant response to DNA virus infection [69,70] and responsible, respectively, for the biogenesis of 22-nt species implicated in secondary sRNA production [71], 24-nt mediating *de novo* DNA methylation [72], and 21-nt involved in PTGS mechanisms [55]. Notably, the 24-nt species have been described as a distinctive feature of nuclear replicating viruses, such as *Geminiviridae* and *Caulimoviridae* [55,69,73,74,75,76], responsible of the methylation of the viral genomic DNA and the regulation of plant antiviral defense [74,77] are evenly distributed along the two viral DNAs. Finally, accumulation of sRNAs along the viral genomic components revealed a hotspot organization, with a higher density of sRNAs on the AC2 gene, which has been also identified as an RNA silencing suppressor in other geminiviral species [78,79] directly involved in the inhibition of the methylation-based defense and a weak PTGS protection [74].

Polarity study of the vd-sRNA showed a roughly equal ratios of positive:negative sRNAs, indicating that although OEGV is a ssDNA virus its sRNAs would be produced from dsRNA precursors derived from sense and antisense strands of the viral genome [69]. Furthermore, analysis of accumulation of OEGV-derived normalized sRNAs (Table 1) suggests a different ability to produce viral derived sRNA and, consequently, a different accumulation of the two genomic components, which appears correlated to the plant genotype (i.e., different cultivar). At tissue level, a general higher accumulation of vd-sRNA has been observed in fruit rather than leaves. This suggests the three cultivars might have intrinsic different level of activity of the silencing machinery components and, that, even remaining constant the accumulation trend of the two genomic components, the activity of PTGS complex is stronger in fruit than in leaf tissues.

In other studies it has been demonstrated that specific AGO proteins can sort sRNAs based on their length and on the nucleotide at the 5′ terminus [80,81,82]. In the case of OEGV, 5′ study of DNA-A mapping-sRNA disclosed a clear prevalence for positive stranded 21-nt with U, which is associated with AGO1, while a prevalence of 5′ A 24-nt in the case of the negative polarity loaded in AGO4. This is coherent with a crucial role of AGO1 in antiviral defense [83]. Conversely, AGO4 binds 24-nt sRNAs formed by DLC3 and it is implicated in DNA methylation and transcriptional gene silencing [84,85,86]. Finally, in the case of DNA-B, a predominance of 22-nt species with C and U at the 5′ termini originating from the positive and negative polarity, respectively, indicates the involvement of AGO5. AGO5 has been described as able to sort specifically 21-, 22- and 24-nt sRNA implicated in PTGS and systemic resistance [87,88].

## 5. Conclusions

In this study, HTS approaches were used to identify a novel species in the *Geminiviridae* family, to characterize its intraspecific diversity and to get insights on the defense mechanisms triggered by this novel geminivirid in olive. To date this is the first circular bipartite ssDNA virus infecting olive. Indeed, although a gemycircularvirus has been recently described on olive, this latter was suggested as a possible virus infecting a fungus associated with olive rather than an olive-infecting virus [89]. Although no correlation of OEGV with a disease or specific symptoms have been found during the survey, we revealed that it is widespread in Apulia, where olive represents one of the most economically and environmentally important crops. Despite the similarities with begomoviruses, sequence comparison, genome organization and phylogenetic analysis were not consistent, and did not endorse allocation of OEGV to any of the genera reported so far, supporting the hypothesis that OEGV could be the first member of a novel genus in the *Geminiviridae* family.

## Figures and Tables

**Table 1:** Small RNA libraries with their OEGV-mapping reads statistics.

	Leucocarpa (LC)		Oliastro (OL)		Toscanina (TS)	
	Leaves	Fruit	Leaves	Fruit	Leaves	Fruit
total	12,340,147	7,813,730	8,218,400	6,545,789	9,488,906	2,968,628
**OEGV mapping reads (raw counts)**						
aligned exactly 1 time	46,810	97,311	23,718	74,586	36,416	35,022
aligned >1 time	2178	4310	1045	5126	1999	3857
total aligned reads	48,988	101,621	24,763	79,712	38,415	38,879
% aligned reads	0.4	1.3	0.3	1.2	0.4	1.3
DNA-A	41,548	91,820	10,655	40,442	17,522	13,942
DNA-B	7440	9801	14,108	39,270	20,893	24,937
**Normalized OEGV mapping reads (RPKM)**						
DNA-A	1213.30	4234.63	467.20	2226.42	665.43	1692.41
DNA-B	218.21	453.97	621.29	2171.29	796.90	3040.24
**Mean coverage of OEGV genomic components (X)**						
DNA-A	324.0	710.9	85.0	318.1	137.4	110.4
DNA-B	59.8	76.6	114.7	311.4	169.1	197.8
**Coverage of OEGV genomic components (% of reference sequence)**						
DNA-A	99.5	99.4	98.2	99.3	99.4	98.5
DNA-B	97.3	98.2	97.9	98.6	98.2	97.3

**Table 2:** Sequence variability among small RNA libraries. SNPs were obtained using the vcftools pipeline.

Sequence	Position	Reference	SNP	Quality Score (Phred)	Genomic Region *
DNA-A	175	T	C	27.08	IR
	239	C	A	109	CP
	673	G	T	51	CP
	725	T	G	30.91	CP
	936	A	C	231	CP
	1021	C	T	258	REn
	1124	C	T	27.71	TrAP/REn
	1160	G	A	59	TrAP/REn
	1259	G	C	69	TrAP/REn
	1350	T	C	20.02	TrAP/REn
	1429	C	T	43.26	TrAP
	1462	G	T	329	TrAP/Rep
	1481	T	C	591	TrAP/Rep
	1761	T	C	106	Rep
	1771	T	C	20.03	Rep
	1915	A	G	273	Rep
	1954	T	G	55	Rep
DNA-B	118	A	T	88	IR
	169	G	A	245	IR
	181	G	A	33.64	IR
	988	C	T	999	MP
	1278	A	T	32.01	Unknown
	2084	G	C	397	IR
	2117	T	C	477	IR
	2178	G	T	24.69	IR

* IR stands for intergenic region; here we are referring to IR to indicate the part of the intergenic region, which falls outside the common region (CR).

## Data Availability

OEGV genomic sequences have been submitted in GenBank under accession numbers MW316657 and MW316658.

## Supplementary Materials

The following are available online at https://www.mdpi.com/1999-4915/13/3/481/s1, Figure S1: Graphical representation of the identity matrix of OEGV with the top 100 complete geminivirid genome hits obtained in BLASTn, Figure S2: Southern blot assay of DNA preparations from OEGV-infected samples hybridized with a specific DNA-A probe. Figure S3: Library and OEGV mapping siRNA size class distribution histograms, Table S1: Table of primers, Table S2: Identity matrix, Table S3: Survey.
