# Supplementary material for: Olea Europaea Geminivirus: A Novel Bipartite Geminivirid Infecting Olive Trees
Source: Viruses. 2021 Mar 15;13(3):481. doi: 10.3390/v13030481 (PMC8000510; doi:10.3390/v13030481)
Supplement: Supplementary file 1 [file viruses-13-00481-s001.zip › Supplementary Figure 2 - Southern Blot.pdf]

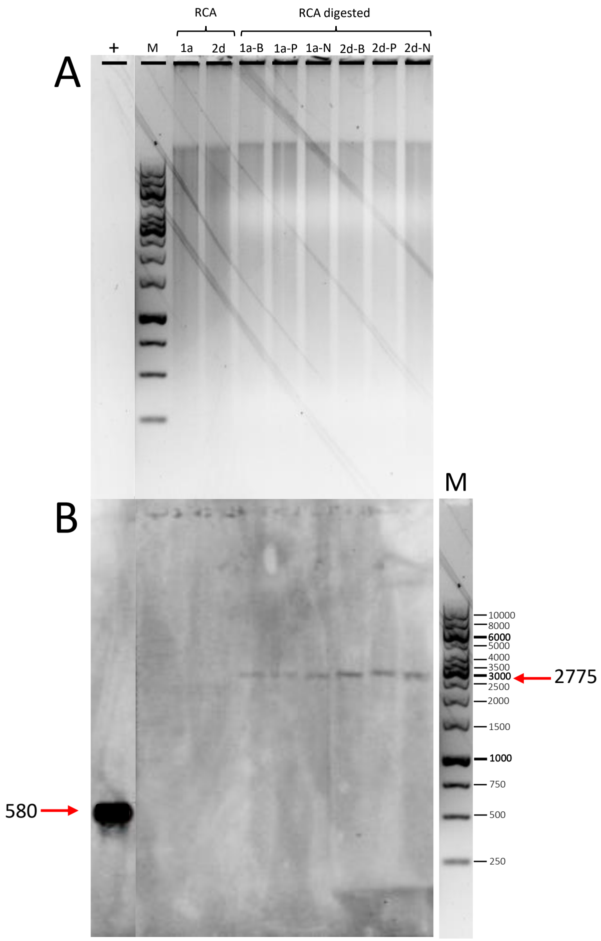

Supplementary Figure 2. Southern blot assay of DNA preparations from OEGV-infected samples hybridized with a specific DNA-A probe. The 1% agarose gel used for the transfer is in the upper panel (A), while in (B) the Southern blot hybridization is showed. Lane 1, DNA-A amplicon used as positive control in a final concentration of 2 ng. Lane 2 (M) GeneRuler 1 kb DNA Ladder (ThermoFisher); lane 3, 4, RCA undigested products obtained from CTAB extracts of samples 1a and 2d; lanes 5-10, RCA products digested with the single cut restriction enzymes Bam-HI (B), PstI (P) and NcoI (N). Red arrows indicate the hybridized 580bp PCR amplicon used as positive control and the linearized 2775 bp full-length DNA-A genomic component.
