# Supplementary material for: Olea Europaea Geminivirus: A Novel Bipartite Geminivirid Infecting Olive Trees
Source: Viruses. 2021 Mar 15;13(3):481. doi: 10.3390/v13030481 (PMC8000510; doi:10.3390/v13030481)
Supplement: Supplementary file 1 [file viruses-13-00481-s001.zip › Supplementary Figure 3 - Library and OEGV mapping siRNA size class distribution histograms.pdf]

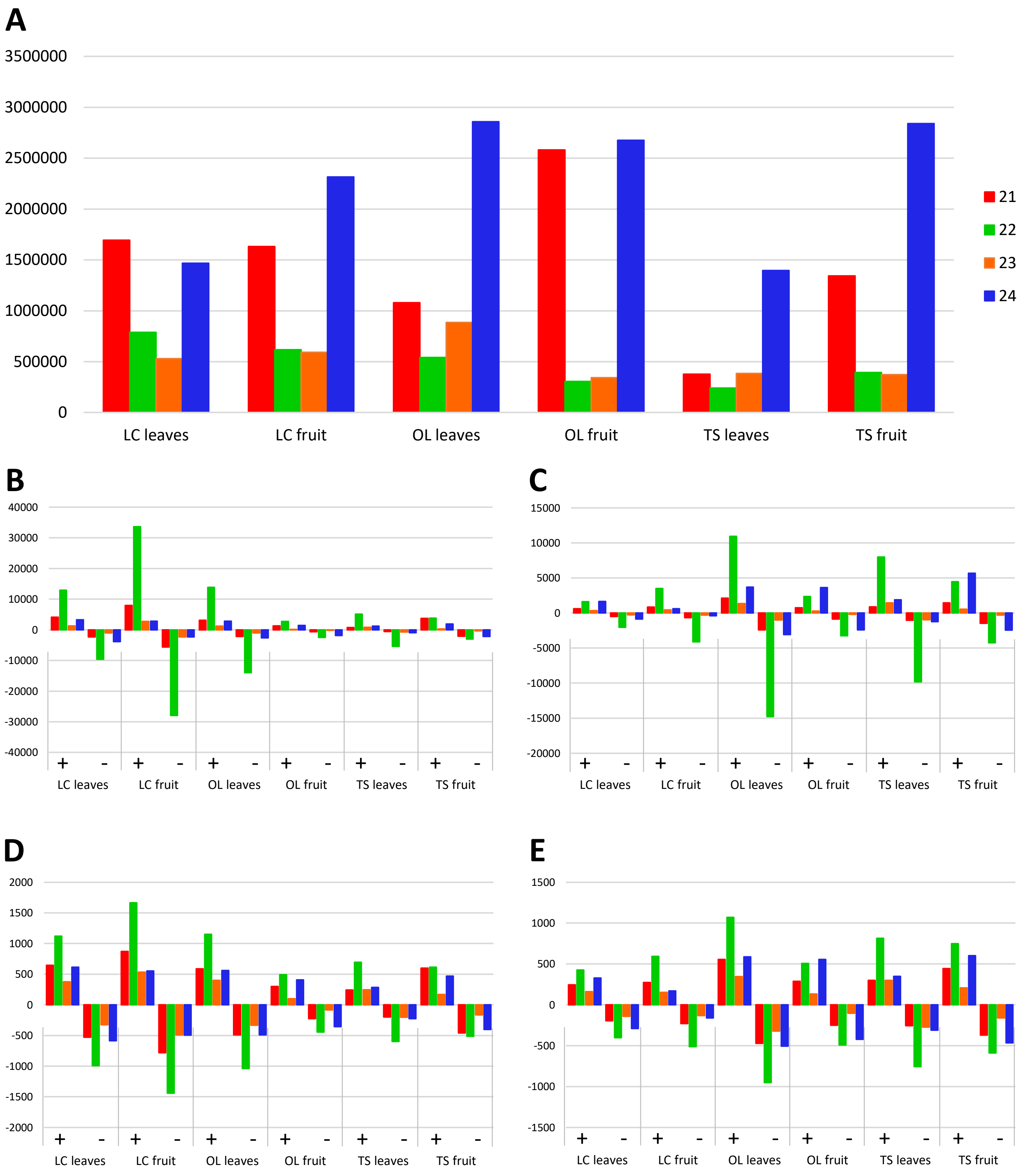

Supplemantary Figure 3. Library profiles by size class distribution profile of 21 to 24 nt redundant smallRNA of the sample analyzed (A). The number of total (B and C) and unique (D and E), sense and antisense vsRNA sequences that map to OEGV DNA A (B and D) and DNA B (C and E). Positive and negative values of the Y-axis, indicate the amount of smallRNAs with positive and negative polarities, respectively.
