# Supplementary material for: Olea Europaea Geminivirus: A Novel Bipartite Geminivirid Infecting Olive Trees
Source: Viruses. 2021 Mar 15;13(3):481. doi: 10.3390/v13030481 (PMC8000510; doi:10.3390/v13030481)
Supplement: Supplementary file 1 [file viruses-13-00481-s001.zip › Supplementary table 1 - Table of Primers.docx]

**Supplementary Table 1**. List of primers used for diagnostic purposes and for resequencing. For each primer, it has been indicated the reference genomic component, the primer name, the sequence (5’-3’), its genomic position and the purpose for which it was used.

| **DNA** | **Primer name** | **Sequence (5’-3’)** | **Position on genome (5’-3’)** | **Notes** |
| --- | --- | --- | --- | --- |
| **DNA-A** | AC1-for* | GGCGTTCATCAGAGTATGTCAGG | 2388-2410 | Diagnostic primer set |
|  | AC1-rev* | GCCTAGAGATCTAGCCCACAGAG | 1831-1853 |  |
| **DNA-B** | BC1-for | GCTGGTCGATCCAGCCAGACAC | 1083-1104 |  |
|  | BC1-rev | GGATCAACATCCTGTCGAAGAGGC | 782-805 |  |
| **DNA-A^#^** | A2for | GGGGACACCTCCGTACGCTTAC | 24-46 | Resequencing |
|  | A3rev | AACAGTAATGGGGACTCCTTCATCTTC | 2525-2551 |  |
|  | A4rev | CTACACTGCCACCAGTGGTGTCC | 833-855 |  |
|  | A5for | GGGCTTACGTCATCTATGATGTTATACC | 1742-1769 |  |
|  | A6for | AGCCGTCGGGATAAAGTCCCAG | 1098-1119 |  |
|  | A7rev | GTGGAAAAACTCTGTGGGCTAGATC | 1838-1862 |  |
| **DNA-B^#^** | B2rev | CCAGATCAGCATTCCCAACAGTGTG | 1477-1501 |  |
|  | B3for | CGGGGACACCTCCGTACGCT | 24-43 |  |
|  | B4for | CGACGTGTTAGAACTGGTTATCTGTGAC | 2134-2161 |  |
|  | B5for | TAATATTACTGGCTTGCCCGCGCC | 1-24 |  |
|  | B7for | CCCACGTTGAACCGTGTGCTTTCG | 856-879 |  |
|  | B8rev | CAACAGTGTGTCAAATGGCGTCG | 1756-1778 |  |
|  | B9for | CAGAGCAGCTCTGAATCGTGCTTC | 1633-1656 |  |
|  | B10rev | CGGACCACCAGAATGACGTCAG | 2361-2382 |  |
|  | B11for | CGACGATATGCGCTGCTGGGAATG | 2251-2274 |  |
|  | B12rev | GGCGCGGGCAAGCCAGTAATATTA | 24-1 |  |

*AC1-for/AC1-rev primers set has been also used to synthesize the probe used for the hybridization assay.

^#^Combination of primers used: DNA-A: A2for/A4rev; A5for/A3rev; A5for/A4rev; A6for/A7rev; A2for/A3rev.

DNA-B: B3for/B2rev, B4for/B2rev, B5for/B2rev, B7for/B8rev, B9for/B10rev, B11for/B12rev.
