# Supplementary figures and images for: Olea Europaea Geminivirus: A Novel Bipartite Geminivirid Infecting Olive Trees
Source: Viruses. 2021 Mar 15;13(3):481. doi: 10.3390/v13030481 (PMC8000510; doi:10.3390/v13030481)

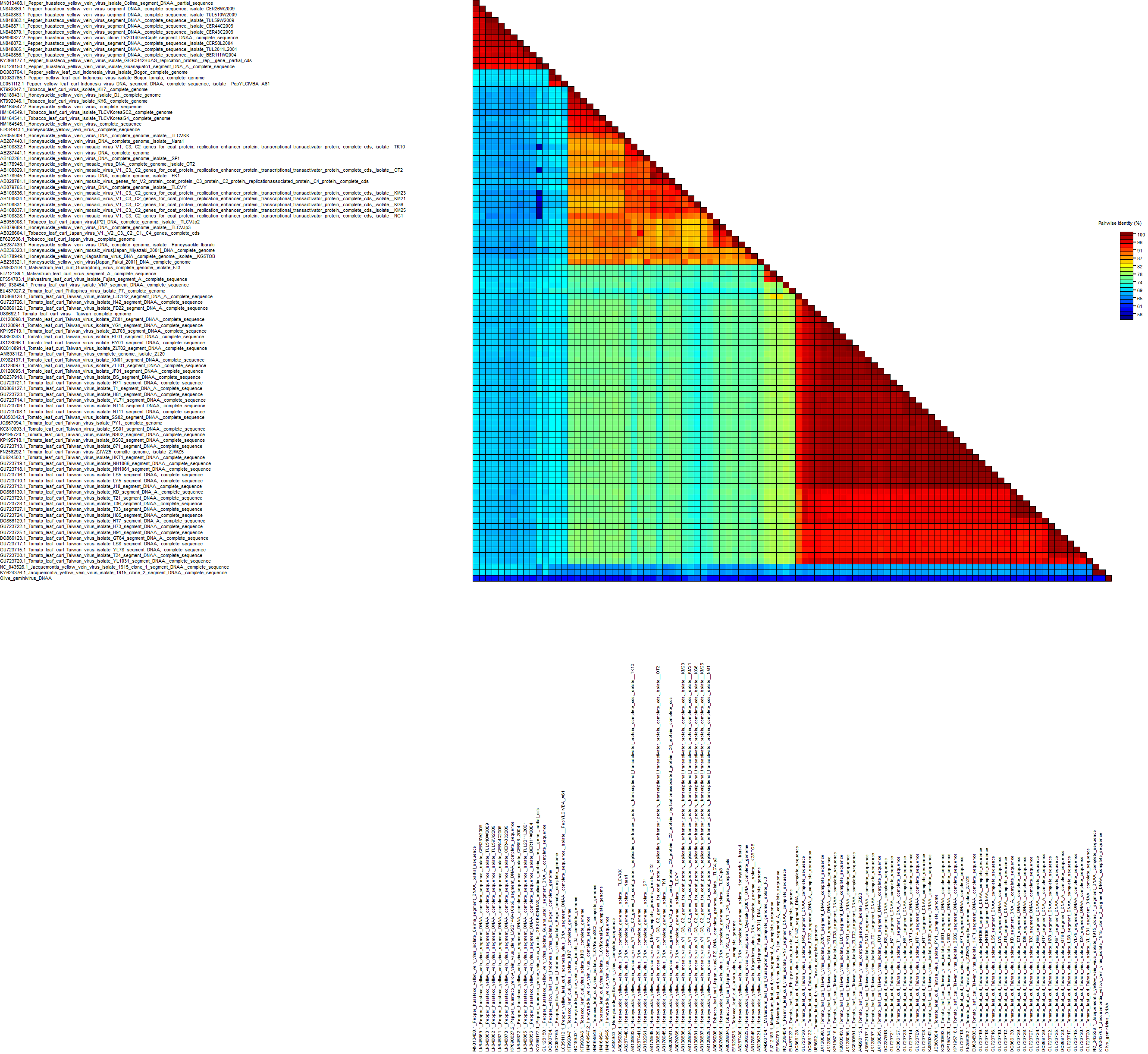

Supplement: Supplementary file 1 [file viruses-13-00481-s001.zip › Supplementary Figure 1 - Graphical representation of the identity matrix of OEGV with the top 100 complete geminivirid genome hits obtained in BLASTn.bmp]
